# A Comparative Assessment of Non-Laboratory-Based versus Commonly Used Laboratory-Based Cardiovascular Disease Risk Scores in the NHANES III Population

**DOI:** 10.1371/journal.pone.0020416

**Published:** 2011-05-31

**Authors:** Ankur Pandya, Milton C. Weinstein, Thomas A. Gaziano

**Affiliations:** 1 Center for Health Decision Science, Harvard School of Public Health, Boston, Massachusetts, United States of America; 2 Department of Health Policy and Management, Harvard School of Public Health, Boston, Massachusetts, United States of America; 3 Divisions of Cardiovascular Medicine, Brigham and Women's Hospital, Boston, Massachusetts, United States of America; University Institute of Social and Preventive Medicine, Switzerland

## Abstract

**Background:**

National and international primary CVD risk screening guidelines focus on using total CVD risk scores. Recently, we developed a non-laboratory-based CVD risk score (inputs: age, sex, smoking, diabetes, systolic blood pressure, treatment of hypertension, body-mass index), which can assess risk faster and at lower costs compared to laboratory-based scores (inputs include cholesterol values). We aimed to assess the exchangeability of the non-laboratory-based risk score to four commonly used laboratory-based scores (Framingham CVD [2008, 1991 versions], and Systematic COronary Risk Evaluation [SCORE] for low and high risk settings) in an external validation population.

**Methods and Findings:**

Analyses were based on individual-level, score-specific rankings of risk for adults in the Third National Health and Nutrition Examination Survey (NHANES III) aged 25–74 years, without history of CVD or cancer (n = 5,999). Risk characterization agreement was based on overlap in dichotomous risk characterization (thresholds of 10-year risk >10–20%) and Spearman rank correlation. Risk discrimination was assessed using receiver operator characteristic curve analysis (10-year CVD death outcome). Risk characterization agreement ranged from 91.9–95.7% and 94.2–95.1% with Spearman correlation ranges of 0.957–0.980 and 0.946–0.970 for men and women, respectively. In men, c-statistics for the non-laboratory-based, Framingham (2008, 1991), and SCORE (high, low) functions were 0.782, 0.776, 0.781, 0.785, and 0.785, with p-values for differences relative to the non-laboratory-based score of 0.44, 0.89, 0.68 and 0.65, respectively. In women, the corresponding c-statistics were 0.809, 0.834, 0.821, 0.792, and 0.792, with corresponding p-values of 0.04, 0.34, 0.11 and 0.09, respectively.

**Conclusions:**

Every score discriminated risk of CVD death well, and there was high agreement in risk characterization between non-laboratory-based and laboratory-based risk scores, which suggests that the non-laboratory-based score can be a useful proxy for Framingham or SCORE functions in resource-limited settings. Future external validation studies can assess whether the sex-specific risk discrimination results hold in other populations.

## Introduction

Cardiovascular disease (CVD) is the leading cause of death globally, with 80% of these deaths occurring in middle and low income countries.[1] Early detection and treatment of individuals at risk is an important strategy for preventing or delaying primary CVD events, thus reducing the health and economic burden of the disease.[2], [3] Most rigorous primary CVD screening guidelines used in developed countries highlight the importance of using absolute CVD or coronary heart disease (CHD) risk scores, such as the Framingham or SCORE (Systematic COronary Risk Evaluation) risk functions, which reflect the combined effects of multiple risk factors on absolute CVD risk.[4]

One challenge of adopting this approach for developing countries is that they not have the cohort studies needed to create and validate their own risk scores. Moreover, they do not have the financial or physical capacity needed to carry out the wide-scale laboratory testing required to implement established laboratory-based risk scores. For example, in India, a cholesterol test that costs $2–4 (U.S. dollars) would account for 5–10% of the 2005 estimate of per capita health spending ($40).[5] With these limitations in mind, the World Health Organization (WHO) and the International Society for Hypertension (ISH) developed separate risk charts that include and exclude laboratory measures (i.e., cholesterol values) for developing world regions. Specifically, the non-laboratory-based charts only require age, sex, smoking status, systolic blood pressure, and diabetes history to estimate total CVD risk. However, the WHO/ISH charts have not yet been validated, nor have they been compared to established laboratory-based scores.[4], [6] If non-laboratory-based risk assessment can be shown to similarly characterize CVD risk compared to laboratory-based approaches, then individual clinicians and national organizations can utilize simple risk scores to serve the same screening function (i.e., identifying high-risk individuals) in a more efficient manner.

Recently, we used the First National Health and Nutrition Examination Survey (NHANES I) to develop a CVD risk score that does not require laboratory inputs (i.e., total and/or HDL cholesterol), which discriminated CVD events as accurately as a total cholesterol-based score.[7] The appeal of a simple CVD risk score is that results are available faster (i.e., all inputs can be obtained within a 5–10 minute office visit) and at less cost relative to risk assessment that requires laboratory testing. While non-laboratory-based risk scores (developed in the NHANES I and Framingham populations) have been shown to predict CVD events well in the cohorts in which they were derived [7], [8], less attention has been given to how these scores compare to laboratory-based scores in external validation populations. Therefore, we sought to assess the exchangeability of the non-laboratory-based score (derived from the NHANES I cohort) to commonly-used laboratory-based scores as they would be used in clinical practice in an external validation population. We conducted our study using data from the Third National Health and Nutrition Examination Survey (NHANES III) population, and found that the non-laboratory-based score characterized and discriminated CVD risk comparably to commonly-used laboratory-based scores.

## Methods

Developing countries are at various stages of the epidemiologic transition, in terms of both the distribution of CVD risk profiles and the progress of implementing CVD prevention and treatment efforts.[9] In order to account for this heterogeneity, we set to compare the non-laboratory-based score to several laboratory-based risks scores that were developed in distinct populations during different time periods. We restricted our analysis to widely-used laboratory-based scores that could be estimated using the variables available in the NHANES III dataset (i.e., age, sex, smoking, history of diabetes, blood pressure treatment, systolic blood pressure, and total and HDL cholesterol). Therefore, we evaluated two versions of Framingham (2008 and 1991 versions) [8], [10] and two versions of SCORE (for high and low risk settings) [11] laboratory-based risk functions, using two methods: 1) We evaluated how similarly the non-laboratory-based risk score characterized individuals in the NHANES III for CVD risk relative to each of the laboratory-based scores; 2) We used follow-up cause-specific mortality data to assess the performance of each risk score in discriminating 10-year CVD death in the NHANES III population.

Appendix S1 describes the study populations that had been used to develop each score, the inputs required for each score, and the composite outcome that each score was designed to predict. Among the laboratory-based risk scores, the composite outcome that was used to construct the Framingham CVD risk equations (myocardial infarction [MI], stroke, congestive heart failure [CHF], CVD death, angina, peripheral vascular disease, coronary insufficiency, and transient ischemic attack) is most similar to the corresponding outcome for the non-laboratory-based risk score (MI, stroke, CHF, CVD death, coronary bypass, percutaneous transluminal coronary angioplasty). We therefore focused on the comparison between the recently developed Framingham (2008) CVD score and the non-laboratory score in our study, although comparisons with the non-laboratory score were performed for all four of the laboratory-based scores.

Although we assessed the performance in risk discrimination of each score based on CVD death in the NHANES III population, we did not alter any of the published coefficients of the scores, even for those designed to predict fatal and non-fatal outcomes (the Framingham and non-laboratory-based risk scores). This allowed us to use the NHANES III follow-up data, which are limited to cause-specific mortality (i.e., no data on non-fatal events), to evaluate the risk scores as they would be used in practice (i.e., based on their published coefficients). It was not possible to assess risk score calibration measures, which focus on predicted versus observed events, for the Framingham and non-laboratory-based scores due to the absence of non-fatal outcomes in the observed events. The time interval for each risk prediction was not relevant (i.e., 10-year or 5-year risk), since all of our analyses were based on score-specific rankings of risk.

### Study Population

The NHANES III is a complex, multi-stage, nationally representative U.S. sample that contains health and nutrition information for 33,394 persons aged 2 months and older.[12] Baseline values were collected from 1988–1994, and cause-specific mortality status is available for adults up to 2006, providing at least 10-year follow-up data for these individuals. Because the sampling for each NHANES was conducted separately, none of the individuals from the NHANES I population were intentionally included in the NHANES III sample. The general methodology and results for the NHANES III are described elsewhere.[13] Among the 20,050 adults in the NHANES III population, 14,973 were between the ages of 25 and 74 years, and 1,742 of these individuals were excluded for history of myocardial infarction, heart failure, stroke or cancer, resulting in 13,248 individuals that met our inclusion criteria. Among these individuals, 5,999 had complete data required to calculate each risk score. Although we focused our study on the population with complete data, we used imputed data to address the possibility of missing values being a confounder in our analysis.

The most common missing variable among individuals who met the inclusion criteria was smoking (missing in 39% and 61% of men and women, respectively), followed by total/HDL cholesterol (missing in 9% and 7% of men and women, respectively). Appendix S2 contains information about missing data for each variable. The NHANES III data files contain five multiple imputation datasets that fill missing data with plausible values using independent draws from predictive distributions, which were generated using multivariate regression methods. The detailed methodology and performance of the NHANES III multiple imputation procedures have been previously reported.[14], [15] Imputed datasets were complete for all variables needed to calculate risk predictions for the five scores included in this study, aside from missing values for history of diabetes for 17 individuals (6 men, 11 women). As a sensitivity analysis, we combined results from multiple imputation datasets (with adjustment for underestimated variance) using methods outlined by Rubin (1987) for scalar (i.e., one-dimensional) estimates.[16] Where possible, results were reported after adjustment for sample weights to account for the complex sampling method used in the NHANES III.[13]

### Statistical analysis

The first step in our analysis was to calculate individual-level risk predictions for each of the five scores included in the study. Individuals were subsequently assigned ranks for each risk score by sex. These ranks were used to assess agreement in dichotomous risk characterization for the non-laboratory-based score compared to each laboratory-based risk score. Using a threshold based on Adult Treatment Panel (APT) III guidelines for treatment of high cholesterol (10-year Framingham CHD risk >10%), individuals were characterized as “high” or “low” risk for each score based on their score-specific rank.[17] An alternative threshold of 10-year CHD risk >20% was also assessed. Percent agreement between the non-laboratory-based score and each of the laboratory-based scores was calculated by adding the proportions of individuals that were equivalently characterized as “high” or “low” risk by both scores. Spearman rank correlation coefficients were computed for the non-laboratory-based score compared to each of the laboratory-based scores to assess the agreement in the rankings across the full spectrum of risk thresholds. Pearson correlation coefficients were not reported because risk score values were not normally distributed.[18]

The performance in risk discrimination for each score was also assessed using the individual score-specific ranks, with 10-year CVD death as the outcome of interest. Causes of death for the NHANES III population are verified by National Death Index (NDI) death certificate match. CVD deaths were defined by having an underlying cause of (International Classification of Diseases [ICD]-10 codes in parentheses): Acute myocardial infarction (I21-I22), other acute ischemic heart disease (I24), atherosclerotic cardiovascular disease (I25.0), all other forms of chronic ischemic heart disease (I20, I25.1–I25.9), or cerebrovascular diseases (I60–I69). As a sensitivity analysis, 10-year total death was also used as a follow-up outcome.

Receiver operator characteristic (ROC) curves were generated for each score, and differences between the areas under each curve (c-statistics) were assessed for the non-laboratory-based score compared to each of the laboratory-based scores. The ROC curve plots the true-positive rate (sensitivity) against the false-positive rate (1-specificity), which graphically depicts the accuracy with which a binary risk classification system (a risk score, in this study) correctly predicts an outcome (10-year CVD death) across the full spectrum of risk thresholds. The c-statistic, or area under the curve (AUC), is a useful single-number summary that quantifies the discriminatory power of the classification system, where values of 1.0 and 0.5 represent perfect and useless systems, respectively.[19] While c-statistics were reported with and without adjustment for sample weights, plots of ROC curves and significance tests for differences in c-statistics were not adjusted for sample weights due to software capability.

Un-weighted and weighted statistical analyses were performed using SAS (Version 9) and SAS Survey Procedures, respectively, and all significance testing was done at the 5% level.[20]

## Results

Table 1 shows the risk profile characteristics of the NHANES III study population by sex for the subpopulation for whom complete data were available, with and without adjustment for sample weights. Appendix S2 shows the same information for the full population, which includes imputed values for missing data. The average clinical characteristics for the imputed, weighted populations skewed towards lower risk profiles compared to the averages for populations with complete, unadjusted data. By design, the weighted NHANES III population is representative of the U.S. population.[21] The linked mortality data files contained follow-up cause-specific death information for >99% of the individuals that met the inclusion criteria. Vital status was known for these individuals for up to 10 years after their baseline interview date.

**Table 1:** Population characteristics of NHANES III population that met inclusion criteria.*

	MEN (n = 3,501)		WOMEN (n = 2,498)	
	Un-weighted	Weighted**	Un-weighted	Weighted**
Age (years)	47.0	44.2	45.6	44.6
Currently smoker	53.8%	52.6%	59.4%	58.3%
History of diabetes	6.5%	4.3%	7.8%	5.5%
Blood pressure treatment	11.1%	8.8%	13.5%	10.0%
Systolic blood pressure (mmHg)	129.1	126.0	122.3	119.2
Total cholesterol (mg/dL)	205.1	204.0	206.5	204.4
HDL cholesterol (mg/dL)	47.4	45.5	54.6	54.4
Body-mass index (kg/m^2^)	26.7	26.6	27.4	26.3
Race	--	--	--	--
White (%)	66.1%	85.2%	64.5%	86.7%
Black (%)	30.5%	10.7%	33.2%	11.4%
Other or unknown (%)	3.4%	4.1%	2.3%	2.0%

*Incluion criteria: 25 yars≤age≤74 years and no history of myocardial infarction, stroke, heart failure, or cancer.

**Data adjusted for complex sampling method used in NHANES III to estimate nationally representative results.

**Figure 1:**
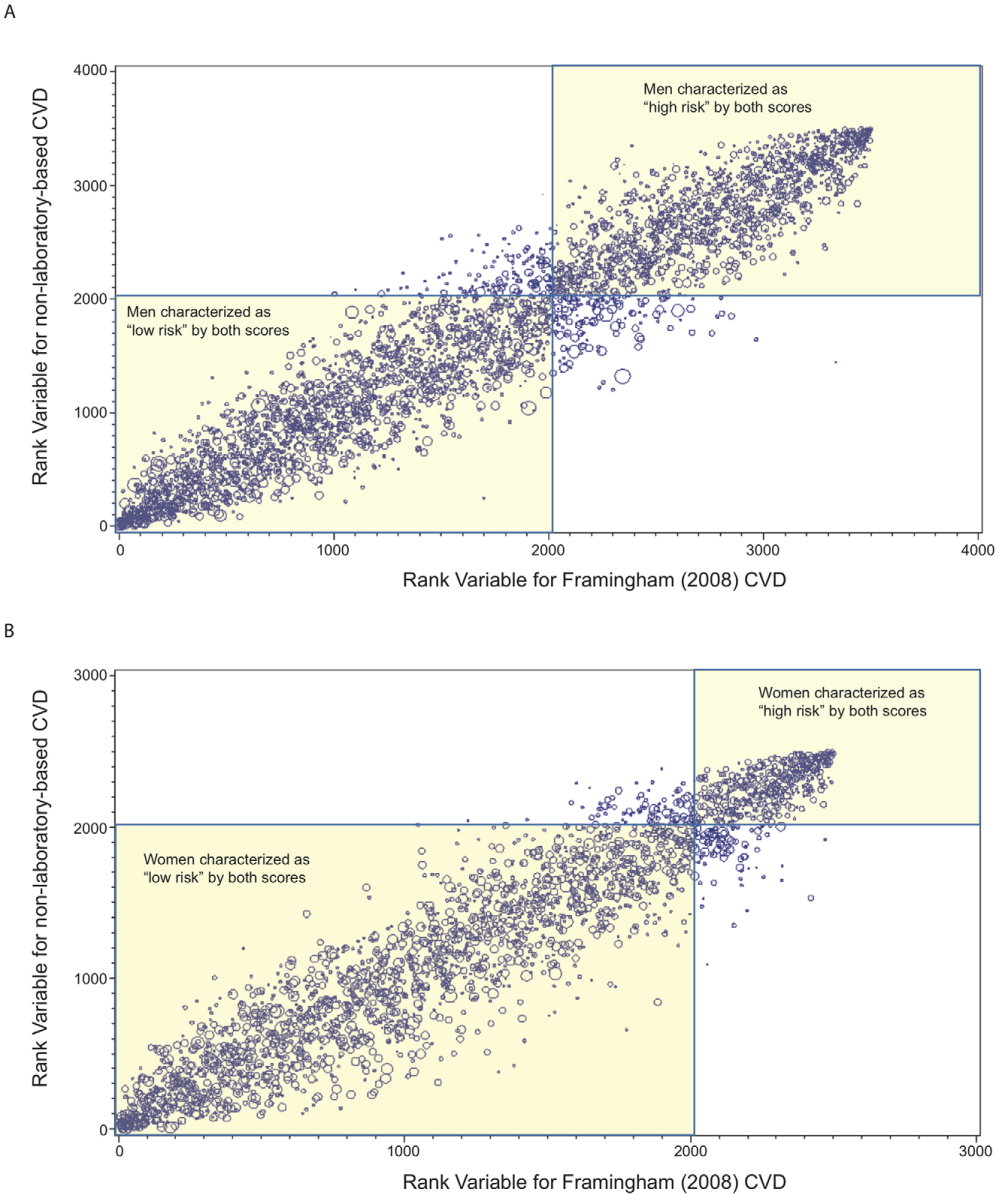
Agreement in risk characterization between Framingham (2008) CVD and non-laboratory-based risk scores. Rank variables for the non-laboratory-based risk score are plotted against rank variables for the Framingham (2008) CVD score for adults aged 25–74 years with complete data in the NHANES III population without history of MI, heart failure, stroke or cancer. Larger ranks indicate greater CVD risk. Size of bubbles correspond to NHANES III sampling weights (i.e., larger bubbles indicate more individuals represented by sample weight). Based on a risk threshold that corresponds to 10-year CHD risk (i.e., top 42.2% of men and 18.8% of women in the sample), 91.9% of men (Panel A) and 94.6% of women (Panel B) would be similarly characterized as high or low risk by the non-laboratory-based and Framingham (2008) CVD risk scores.

Based on a risk threshold that corresponds with a 10-year CHD risk >10%, 42.2% of men and 18.8% of women in the study sample (with complete data) would be characterized as “high” risk. Figures 1a and 1b show the ranks for CVD risk as assessed by the non-laboratory-based score plotted against the ranks for CVD based on the Framingham (2008) CVD risk equation for men and women, respectively. The figures show that 91.9% of men and 94.6% of women would be characterized as “high” or “low” risk consistently using either risk score. Equivalently, 8.1% of men and 5.4% of women would be re-characterized as “high risk” by one score to “low risk” by the other or vice versa. Table 2 shows the percent agreement between the non-laboratory-based and all of the laboratory-based scores using the same threshold. Agreement was similar across genders, completeness of data, and adjustment of sample weights. Percent agreement ranged from 91.9–95.7% and 94.2–95.1% across the laboratory-based scores for men and women, respectively. When a threshold of 10-year CHD risk >20% was used (i.e., the top 16.8% and 4.2% of men and women in the study sample would be characterized as “high” risk, respectively), the corresponding agreement ranges were 94.9–96.5% and 96.6–97.9% for men and women, respectively. Appendix S3 shows that these trends were consistent when the full population (with imputed values) was analyzed.

**Table 2:** Risk categorization results for four laboratory-based risk scores, each compared to non-laboratory-based risk score.

MEN (limited to those with complete data, n = 3,501)			
Score	Un-weighted agreement*	Weighted agreement*	Spearman correlation**
Framingham CVD (2008)	92.2%	91.9%	0.957
Framingham CVD (1991)	92.5%	91.9%	0.961
SCORE high risk	95.6%	95.3%	0.979
SCORE low risk	95.8%	95.7%	0.980

WOMEN (limited to those with complete data, n = 2,498)			
Framingham CVD (2008)	93.4%	94.6%	0.946
Framingham CVD (1991)	92.8%	94.2%	0.946
SCORE high risk	94.3%	95.1%	0.970
SCORE low risk	94.4%	95.1%	0.970

*“Agreement” based on dichotomous risk categorization corresponding to 10-year Framingham CHD risk >10%.

**All p-values for Spearman rank correlation coefficients <0.0001.

**Spearman correlation results only available for un-weighted populations.

Table 2 also shows the Spearman correlation coefficients for each of the laboratory-based risk scores compared to the non-laboratory risk score. The Spearman correlation results were similar to the percent agreement findings in terms of trends across scores and between imputed versus non-imputed datasets. Specifically, the Spearman rank correlation coefficients ranged from 0.957–0.980 and 0.946–0.970 across the laboratory-based scores for men and women, respectively. All of the p-values for the Spearman rank correlation coefficients were <0.001, even after adjusting the variance of imputed datasets. Appendix S3 shows that the Spearman correlation results were consistent when the full population (with imputed values) was analyzed.

**Figure 2:**
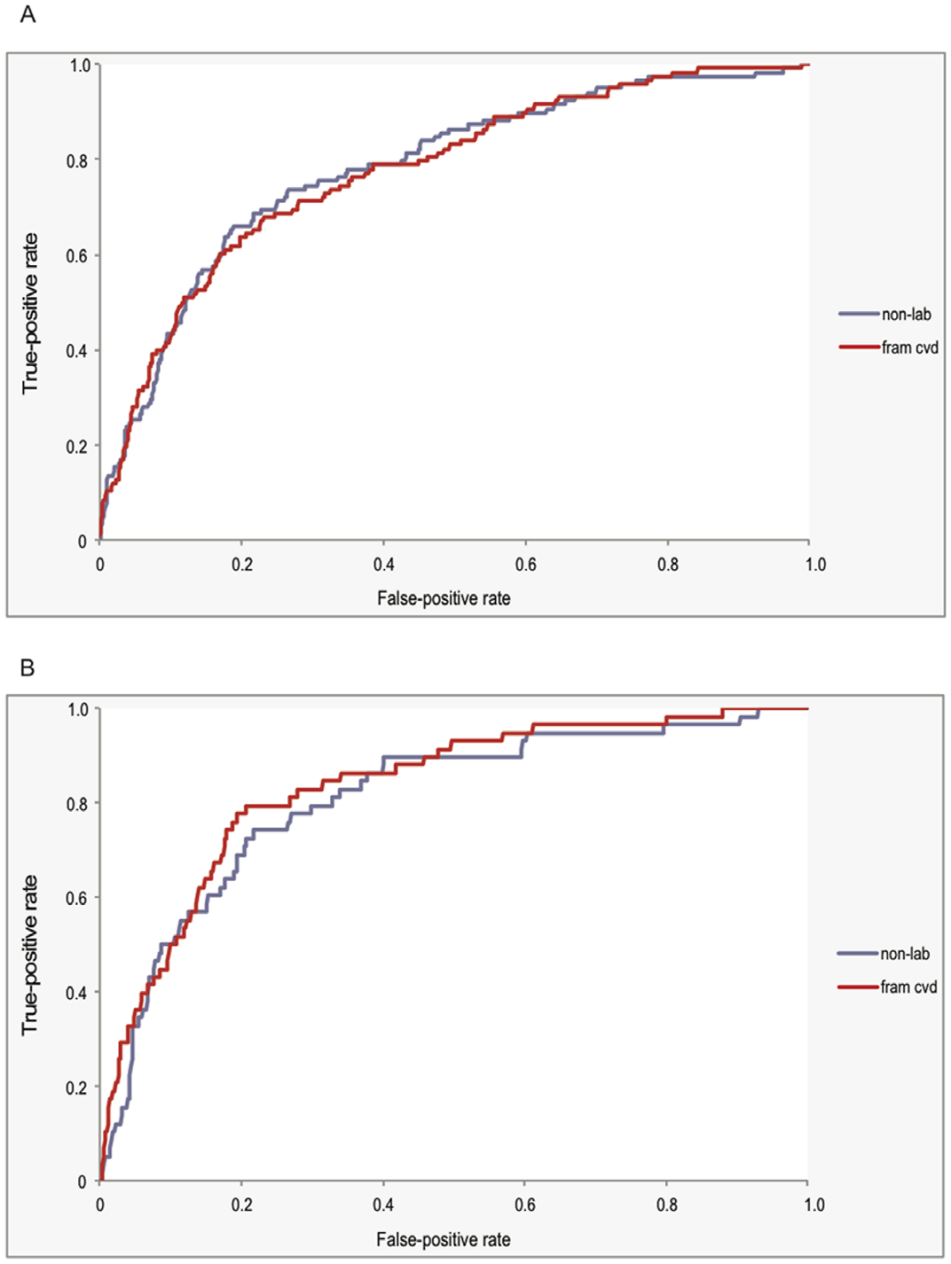
ROC curves (10-year CVD death outcome) for non-laboratory-based and Framingham (2008) CVD risk scores. Receiver operator characteristic (ROC) curves for the non-laboratory-based (“non-lab”) and Framingham (2008) CVD (“fram cvd”) scores, with 10-year CVD death as the outcome of interest, for individuals with complete data. For men (Panel A), the performances in risk discrimination, as assessed by the c-statistic (i.e., area under the ROC curve) and 95% CI, were 0.782 (0.739–0.825) and 0.776 (0.733–0.819) for the non-laboratory-based and Framingham (2008) CVD risk scores, respectively, with a p-value for the difference of 0.44. For women (Panel B), the c-statistics and 95% CI were 0.809 (0.751–0.866) and 0.834 (0.782–0.885) for the non-laboratory-based and Framingham (2008) CVD risk scores, respectively, with a p-value for the difference of 0.04.

From 10-year follow-up data for each individual (excluding those with imputed risk characteristics values), there were 118 and 58 CVD deaths for men and women, which represented 26.6% and 25.3% of the total deaths within the 10-year follow-up period, respectively. Figures 2a and 2b show the ROC curves for the non-laboratory-based risk score and the Framingham (2008) CVD risk equation for men and women, respectively. For men (Figure 2a), the c-statistics and 95% confidence intervals (CIs) for the non-laboratory-based and Framingham (2008) CVD scores were 0.782 (0.739–0.825) and 0.776 (0.733–0.819), respectively. The corresponding c-statistics and 95% CIs for women (Figure 2b) were 0.809 (0.751–0.866) and 0.834 (0.782–0.885), respectively. The differences between the non-laboratory-based and the Framingham (2008) CVD scores were not statistically significant for men, but were for women (p-values of 0.44 and 0.04, respectively). These results were similar after adjusting for sample weights (c-statistics of 0.782 and 0.772 for the non-laboratory-based and Framingham CVD risk scores in men, and 0.807 and 0.832 in women, respectively). Appendix S4 shows that these trends were consistent for the full population (with imputed values), for both weighted and un-weighted analyses.

Table 3 and Appendix S4 also show the c-statistics with 95% CIs for all scores by sex, completeness of data, and with and without adjustment for sample weights. As seen in these tables, the non-laboratory-based score did not have a statistically significantly different c-statistics in any of the comparisons to the four laboratory-based scores for men. In women, the non-laboratory-based score had statistically significantly lower c-statistics compared to the Framingham (2008) CVD risk function (in the populations with and without imputed data), and statistically higher c-statistics compared to the SCORE functions (for high and low risk settings) in the full population that contained imputed data (p-values of 0.02 and 0.01, respectively). Applying sample weights resulted in very similar differences in c-statistics between the non-laboratory-based and laboratory-based scores, relative to un-weighted analyses. The non-laboratory-based score had higher c-statistics compared to all laboratory-based scores in sensitivity analyses using 10-year total death instead of 10-year CVD death as the outcome of interest.

**Table 3:** Risk discrimination results for four laboratory-based risk scores, each compared to non-laboratory-based risk score–CVD death.

MEN (limited to those with complete data, n = 3,501)							
score	c-statistic (95% CI), un-weighted	p-value*	c-statistic, weighted**	sensitivity***	specificity***	PPV***	NPV***
Non-laboratory-based	0.782 (0.739, 0.825)	--	0.782	0.788	0.592	0.063	0.988
Framingham CVD (2008)	0.776 (0.733, 0.819)	0.44	0.772	0.788	0.591	0.063	0.988
Framingham CVD (1991)	0.781 (0.738, 0.823)	0.89	0.778	0.780	0.591	0.063	0.987
SCORE high risk	0.785 (0.743, 0.826)	0.68	0.784	0.805	0.592	0.065	0.989
SCORE low risk	0.785 (0.743, 0.826)	0.65	0.785	0.814	0.592	0.065	0.989

WOMEN (limited to those with complete data, n = 2,498)							
Non-laboratory-based	0.809 (0.751, 0.866)	--	0.807	0.638	0.823	0.079	0.990
Framingham CVD (2008)	0.834 (0.782, 0.885)	*0.04*	0.832	0.707	0.825	0.087	0.992
Framingham CVD (1991)	0.821 (0.766, 0.876)	0.34	0.821	0.700	0.824	0.085	0.991
SCORE high risk	0.792 (0.731, 0.854)	0.11	0.793	0.638	0.823	0.079	0.990
SCORE low risk	0.792 (0.730, 0.854)	0.09	0.792	0.655	0.823	0.081	0.990

*Difference in c-statistic compared to non-laboratory-based score, using un-weighted data.

*Italics indicate p-value <0.05.

**Standard errors not available for weighted results.

***Using 10-year CHD risk >10% threshold, un-weighted data.

Abbreviations: Positive predictive value (PPV), negative predictive value (NPV).

## Discussion

In this study, we assessed the extent to which a non-laboratory-based CVD risk score similarly ranked individuals and discriminated risk of CVD death compared to four versions of laboratory-based Framingham and SCORE equations. We observed strong agreement in risk characterization between the non-laboratory-based and laboratory-based scores, and that all scores performed well in discriminating 10-year risk of CVD death in an external validation cohort (the NHANES III population). Over 91% of men and 94% of women were equivalently characterized as “high” or “low risk” by non-laboratory-based and laboratory-based scores using a risk threshold based on current U.S. guidelines (10-year CHD risk >10%).[17] This finding was robust under a stricter risk threshold (10-year CHD risk >20%) and across the full spectrum of risk. The results varied by sex for the risk discrimination comparisons. In men, there were no statistical differences in the prediction of 10-year CVD death for the non-laboratory-based risk score compared to any of the laboratory-based scores. In women, the recent Framingham (2008) CVD score had statistically higher c-statistic (0.834) compared to the non-laboratory-based score (0.809). All of the findings were consistent when analyses were conducted with and without imputed data and sample weights.

A potential problem of using any absolute CVD risk assessment tool is that the population in which a given score was derived may or may not be appropriate for the population to which it is ultimately applied. Ideally, more cohort studies would be conducted to develop setting-specific risk scores. However, since many developing countries do not have the resources to conduct a representative cohort study and develop their own scores, they have little choice but to use scores that were derived from other (more developed) settings as they have been doing for the last two decades. Certainly, established risk scores from developed regions may over- or underestimate risk in a given population. But if the risk scores rank individuals similarly, a country could choose a risk threshold to identify a certain percentage of its population that it could afford to manage aggressively with medical therapy, while offering life-style interventions for those at lower risk and population-based strategies to reduce the risk in the overall population. This approach would be consistent with previous cost-effectiveness studies that have shown that treatment decisions based on the aggregate effects of multiple risk factors can result in more efficient outcomes compared to treatment guidelines based on single risk factors.[22], [23], [24] In our analysis, there was a high level of agreement among the scores in stratifying individuals into high and low risk categories, which suggests that a non-laboratory-based risk score could reasonably serve the same screening function as more expensive laboratory-based risk scores in resource-poor settings.

The second objective of this study was to assess the risk discrimination performance of each score in an external validation population, which, to our knowledge, has not been evaluated for non-laboratory-based CVD risk scores. We found that all scores in analysis performed well, with c-statistics greater than 0.77. A recent review of risk scores found that only 5 out of 17 external validations of the Framingham and SCORE equations had c-statistics greater than 0.77.[4] Future studies can examine if the sex-specific trends we found are observed in other cohorts. If scores behave similarly in men, attention should be given to the most convenient or least costly scores when updating or creating primary CVD screening guidelines for developing and developed countries. One example of how non-laboratory-based risk scores could be incorporated was proposed in a recent modeling study by Chamnam et al., where simple risk assessment was included as the first stage of a stepwise screening strategy.[25] The differences in risk discrimination between scores in women could be due to differential biological effects for specific risk factors, such as cholesterol (for the differences between the non-laboratory-based and Framingham scores) or diabetes and treatment for hypertension (for the differences between the SCORE functions and all other scores), or these findings could be due to statistical chance (p-value for difference between recent Framingham CVD and non-laboratory-based score = 0.04). More research investigating these sex-specific differences could further explain these findings.

A limitation of our analysis was that the follow-up data were restricted to cause-specific mortality outcomes, which prevented the calculation of risk score calibration measures. Although calibration is an important component of risk score assessment, scores are often recalibrated for different settings, a process that does not alter the β-coefficients of the risk scores.[4], [26] Therefore, our findings would not be affected if scores are recalibrated, since rankings of risk are only dependent on risk score β-coefficients. However, as mentioned by Panagiotakos and Stavrinos, recalibration would require representative CVD incidence data, which could be limited depending on the setting.[27]

In addition to the lack of calibration, our analysis did not include the commonly-used ASSIGN-SCORE, PROCAM and QRISK functions, as the social deprivation and family history variables needed to calculate these functions were not available in the NHANES III data.[28], [29], [30] The WHO/ISH risk charts were not included because the underlying risk functions are not publicly available.[6] Our main risk discrimination results were also based on a relatively small number of events (176 CVD deaths). Future external validation studies could be designed to address these limitations.

In summary, we found that the non-laboratory-based and laboratory-based scores evaluated in this study discriminated risk of CVD death well in this external validation cohort. In men, the non-laboratory-based score discriminated risk similarly to all of the laboratory-based scores, which could have implications for developing efficient, sex-specific primary CVD screening guidelines. Further investigation of the risk discrimination and calibration performance of the non-laboratory-based score could help confirm this finding. However, even if it assumed that laboratory-based risk assessment is the gold standard, we found that the non-laboratory-based risk score similarly characterized high and low risk men and women compared to the Framingham and SCORE functions. In resource-poor settings, non-laboratory-based risk assessment could serve as a useful proxy for these more intensive, expensive risk screening approaches.

## Supporting Information

Appendix S1 **Risk scores calculated for adults in NHANES III population (1988–1994).**
(DOC)

Appendix S2 **Population characteristics of NHANES III population (including those with imputed values) that met inclusion criteria.**
(DOC)

Appendix S3 **Risk categorization results for four laboratory-based risk scores, each compared to non-laboratory-based risk score.**
(DOC)

Appendix S4 **Risk discrimination results for four laboratory-based risk scores, each compared to non-laboratory-based risk score-CVD death.**
(DOC)

## References

[1] Murray CJ, Lopez AD (1997). Mortality by cause for eight regions of the world: Global Burden of Disease Study.. Lancet.

[2] Services  DoHaH (2000). Healthy People 2010: Understanding and Improving Health..

[3] Jackson R, Lynch J, Harper S (2006). Preventing coronary heart disease.. BMJ.

[4] Cooney MT, Dudina A, D'Agostino R, Graham IM (2010). Cardiovascular risk-estimation systems in primary prevention: do they differ? Do they make a difference? Can we see the future?. Circulation.

[5] Bank W (2007). World development indicators.. World Bank.

[6] World Health Organization (2007). Prevention of cardiovascular disease.. Pocket guidelines for assessment and management of cardiovascular risk.

[7] Gaziano TA, Young CR, Fitzmaurice G, Atwood S, Gaziano JM (2008). Laboratory-based versus non-laboratory-based method for assessment of cardiovascular disease risk: the NHANES I Follow-up Study cohort.. Lancet.

[8] D'Agostino RB, Vasan RS, Pencina MJ, Wolf PA, Cobain M (2008). General cardiovascular risk profile for use in primary care: the Framingham Heart Study.. Circulation.

[9] Mendis S, Abegunde D, Yusuf S, Ebrahim S, Shaper G (2005). WHO study on Prevention of REcurrences of Myocardial Infarction and StrokE (WHO-PREMISE).. Bulletin of the World Health Organization.

[10] Anderson KM, Odell PM, Wilson PW, Kannel WB (1991). Cardiovascular disease risk profiles.. Am Heart J.

[11] Conroy RM, Pyorala K, Fitzgerald AP, Sans S, Menotti A (2003). Estimation of ten-year risk of fatal cardiovascular disease in Europe: the SCORE project.. Eur Heart J.

[12] National Health and Nutrition Examination Survey (2011). Centers for Disease Control and Prevention (CDC).. National Center for Health Statistics (NCHS).

[13] Vital Health (1994). Plan and operation of the Third National Health and Nutrition Examination Survey, 1988-94. Series 1: programs and collection procedures.. Vital Health Stat.

[14] Raghunathan TE, Lepkowskil JM, Van Hoewyk J (2001). A Multivariate Technique for Multiply Imputing Missing Values Using a Sequence of Regression Models.. Survey Methodology.

[15] Little RJA, Ezzati TM, Johnson W, Khare M, Rubin DM (1995). A simulation study to evaluate the performance of model-based multiple imputations in NCHS health examination surverys..

[16] Rubin DB (1987). Multiple Imputation for Nonresponse in Surveys..

[17] NCEP (2002). Third Report of the National Cholesterol Education Program (NCEP) Expert Panel on Detection, Evaluation, and Treatment of High Blood Cholesterol in Adults (Adult Treatment Panel III) final report.. Circulation.

[18] Gaddis ML, Gaddis GM (1990). Introduction to biostatistics: Part 6, Correlation and regression.. Ann Emerg Med.

[19] Pencina MJ, D'Agostino RB (2004). Overall C as a measure of discrimination in survival analysis: model specific population value and confidence interval estimation.. Stat Med.

[20] Baisden KL, Hu P. The Engima of Survey Data Analysis: Comparison of SAS Survey Procedures and SUDAAN Procedures; 2006; Cary, NC.. SAS Institute.

[21] Ezzati TM, Massey JT, Waksberg J, Chu A, Maurer KR (1992). Sample design: Third National Health and Nutrition Examination Survey.. Vital Health Stat.

[22] Prosser LA, Stinnett AA, Goldman PA, Williams LW, Hunink MG (2000). Cost-effectiveness of cholesterol-lowering therapies according to selected patient characteristics.. Ann Intern Med.

[23] Gaziano TA, Steyn K, Cohen DJ, Weinstein MC, Opie LH (2005). Cost-effectiveness analysis of hypertension guidelines in South Africa: absolute risk versus blood pressure level.. Circulation.

[24] Gaziano TA, Opie LH, Weinstein MC (2006). Cardiovascular disease prevention with a multidrug regimen in the developing world: a cost-effectiveness analysis.. Lancet.

[25] Chamnan P, Simmons RK, Khaw KT, Wareham NJ, Griffin SJ (2010). Estimating the population impact of screening strategies for identifying and treating people at high risk of cardiovascular disease: modelling study.. BMJ.

[26] D'Agostino RB, Grundy S, Sullivan LM, Wilson P, Grp CRP (2001). Validation of the Framingham Coronary Heart Disease prediction scores-Results of a multiple ethnic groups investigation.. Jama-Journal of the American Medical Association.

[27] Panagiotakos DB, Stavrinos V (2006). Methodological issues in cardiovascular epidemiology: the risk of determining absolute risk through statistical models.. Vasc Health Risk Manag.

[28] Hippisley-Cox J, Coupland C, Vinogradova Y, Robson J, May M (2007). Derivation and validation of QRISK, a new cardiovascular disease risk score for the United Kingdom: prospective open cohort study.. BMJ.

[29] Assmann G, Cullen P, Schulte H (2002). Simple scoring scheme for calculating the risk of acute coronary events based on the 10-year follow-up of the prospective cardiovascular Munster (PROCAM) study.. Circulation.

[30] Woodward M, Brindle P, Tunstall-Pedoe H (2007). Adding social deprivation and family history to cardiovascular risk assessment: the ASSIGN score from the Scottish Heart Health Extended Cohort (SHHEC).. Heart.

