# Supplementary material for: A Comparative Assessment of Non-Laboratory-Based versus Commonly Used Laboratory-Based Cardiovascular Disease Risk Scores in the NHANES III Population
Source: PLoS One. 2011 May 31;6(5):e20416. doi: 10.1371/journal.pone.0020416 (PMC3105026; doi:10.1371/journal.pone.0020416)
Supplement: Appendix S1 — Risk scores calculated for adults in NHANES III population (1988–1994). (DOC) [file pone.0020416.s001.doc]

| Appendix S1. Risk scores calculated for adults in NHANES III population (1988-1994)   | Variable (and beta coefficients)* | Framingham CVD 2008 (D'Agostino et al., 2008)^8^ | Framingham CVD 1991 (Anderson et al., 1991)^10^ | SCORE, high risk (Conroy et al., 2003)^11^ | SCORE, low risk (Conroy et al., 2003)^11^ | Non-laboratory-based (Gaziano et al., 2008)^7^ | | --- | --- | --- | --- | --- | --- | | Population (years for baseline values) | Framingham, MA, U.S. (1968-1987) | Framingham, MA, U.S. (1968-1975) | High risk European countries** (1972-88) | Low risk European countries*** (1972-88) | NHANES I (U.S., 1971-75) | | Age | 3.061, 2.329 | several forms**** | Included***** | Included***** | 3.560, 3.904 | | Sex | sex-specific predictions | 18.814 | Included***** | Included***** | sex-specific predictions | | Smoking | 0.655, 0.529 | -0.390 | Included***** | Included***** | 0.568, 0.571 | | Diabetes | 0.574, 0.692 | -0.304, -0.473 | Not included | Not included | 0.641, 0.649 | | SBP | 1.933, 2.762 | -1.403 | Included***** | Included***** | 1.627, 1.545 | | Total cholesterol | 1.124, 1.209 | total/HDL ratio: -0.539 | Included***** | Included***** | Not included | | HDL cholesterol | -0.933, -0.708 | total/HDL ratio: -0.539 | Not included | Not included | Not included | | Treatment of hypertension | changes coefficients for SBP to: 1.999, 2.823 | Not included | Not included | Not included | 0.220, 0.367 | | BMI | Not included | Not included | Not included | Not included | 0.727, 0.847 | | Outcome | MI, angina, coronary insufficiency, CHD death, stroke, TIA, CHF, PVD, CVD death | Same as Framingham CVD 2008 | Death from: hypertensive disease, IHD, cerebrovascular disease | Same as SCORE, high risk | CVD death, MI, stroke, CHF, coronary bypass, PTCA | | *Beta coefficient listed (for men, women), if included in risk score inputs, and refer to natural logs for continuous variables for references 8, 10, and 7 | | | | | | | **Applicable for all non-low risk European countries | | | | | | | ***Applicable for Belgium, France, Greece, Italy, Luxembourg, Portugal, Spain, and Switzerland | | | | | | | ****coefficient for (log(age)): -1.215, coefficient for (log (age^2^)): -1.844, coefficient for (log(age^2^)*female): 0.367 | | | | | | | *****specific risk factor coefficients not displayed due to complex equation | | | | | | | Abbreviations: National Health and Nutrition Examination Survey (NHANES), high-density lipoprotein (HDL), body-mass index (BMI), myocardial infarction (MI), coronary heart disease (CHD), transient ischemic attack (TIA), congestive heart failure (CHF), peripheral vascular disease (PVD), cardiovascular disease (CVD), ischemic heart disease (IHD), percutaneous transluminal coronary angioplasty (PTCA), systolic blood pressure (SBP) | | | | | | | | |
| --- | --- | --- | --- | --- | --- | --- | --- | --- | --- | --- | --- | --- | --- | --- | --- | --- | --- | --- | --- | --- | --- | --- | --- | --- | --- | --- | --- | --- | --- | --- | --- | --- | --- | --- | --- | --- | --- | --- | --- | --- | --- | --- | --- | --- | --- | --- | --- | --- | --- | --- | --- | --- | --- | --- | --- | --- | --- | --- | --- | --- | --- | --- | --- | --- | --- | --- | --- | --- | --- | --- | --- | --- | --- | --- | --- | --- | --- | --- | --- | --- | --- | --- | --- | --- | --- | --- | --- | --- | --- | --- | --- | --- | --- | --- | --- | --- | --- | --- | --- | --- | --- | --- | --- | --- | --- | --- | --- | --- |
