# Supplementary material for: A Comparative Assessment of Non-Laboratory-Based versus Commonly Used Laboratory-Based Cardiovascular Disease Risk Scores in the NHANES III Population
Source: PLoS One. 2011 May 31;6(5):e20416. doi: 10.1371/journal.pone.0020416 (PMC3105026; doi:10.1371/journal.pone.0020416)
Supplement: Appendix S2 — Population characteristics of NHANES III population (including those with imputed values) that met inclusion criteria. (DOC) [file pone.0020416.s002.doc]

| Appendix S2. Population characteristics of NHANES III population (including those with imputed values) that met inclusion criteria* | | | | | | | |
| --- | --- | --- | --- | --- | --- | --- | --- |
|  |  |  |  |  |  |  |  |
|  | MEN (full population, n=6,273) | | | WOMEN (full population, n=6,958) | | |  |
|  | % missing | Un-weighted | Weighted** | % missing | Un-weighted | Weighted** |  |
| Age (years) | 0.0% | 45.5 | 43.3 | 0.0% | 45.5 | 44.7 |  |
| Currently smoker | 39.1% | 32.8% | 31.8% | 61.0% | 23.1% | 26.1% |  |
| History of diabetes | 0.1% | 5.3% | 3.7% | 0.2% | 7.8% | 4.8% |  |
| Blood pressure treatment | 0.0% | 10.1% | 7.7% | 0.0% | 13.2% | 9.9% |  |
| Systolic blood pressure (mmHg) | 0.1% | 127.4 | 124.6 | 0.1% | 122.0 | 118.7 |  |
| Total cholesterol (mg/dL) | 8.6% | 201.8 | 200.5 | 7.3% | 203.9 | 202.4 |  |
| HDL cholesterol (mg/dL) | 8.6% | 47.3 | 45.7 | 7.3% | 54.7 | 55.2 |  |
| Body-mass index (kg/m^2^) | 8.6% | 26.7 | 26.6 | 7.3% | 27.6 | 26.4 |  |
| Race | 0% | -- | -- | 0% | -- | -- |  |
| White (%) | -- | 65.7% | 84.3% | -- | 64.6% | 83.0% |  |
| Black (%) | -- | 30.6% | 11.0% | -- | 32.1% | 12.6% |  |
| Other or unknown (%) | -- | 3.8% | 4.7% | -- | 3.3% | 4.4% |  |
| **Inclusion criteria: 25*  *years ≤ age ≤ 74 years, and no history of myo*  *ardial*  *infarction, stroke, heart failure, or cancer* | | | | | | |  |
| ***Data adjusted*  *or complex sampling method used in NHANES III to estimate nationally representative results* | | | | | | |  |
