# Supplementary material for: A Comparative Assessment of Non-Laboratory-Based versus Commonly Used Laboratory-Based Cardiovascular Disease Risk Scores in the NHANES III Population
Source: PLoS One. 2011 May 31;6(5):e20416. doi: 10.1371/journal.pone.0020416 (PMC3105026; doi:10.1371/journal.pone.0020416)
Supplement: Appendix S3 — Risk categorization results for four laboratory-based risk scores, each compared to non-laboratory-based risk score. (DOC) [file pone.0020416.s003.doc]

Appendix S3. Risk categorization results for four laboratory-based risk scores, each compared to non-laboratory-based risk score

| MEN (full population, n=6,273) | | | |
| --- | --- | --- | --- |
| score | Un-weighted agreement* | Weighted agreement* | Spearman correlation** |
| Framingham CVD (2008) | 93.4% | 93.6% | 0.962 |
| Framingham CVD (1991) | 93.4% | 93.5% | 0.966 |
| SCORE high risk | 96.2% | 96.3% | 0.984 |
| SCORE low risk | 96.4% | 96.4% | 0.984 |
| WOMEN (full population, n=6,958) | | | |
| Framingham CVD (2008) | 94.4% | 95.2% | 0.951 |
| Framingham CVD (1991) | 93.8% | 94.8% | 0.954 |
| SCORE high risk | 94.3% | 95.7% | 0.974 |
| SCORE low risk | 94.3% | 95.5% | 0.973 |
| **“Agreement” based on dichotomous risk categorization corresponding to 10-year Framingham CHD risk >10%* | | | |
| ***All p-values for Spearman rank correlation coefficients <0.0001* | | | |
| ***Spearman correlation results only available for un-weighted populations* | | | |
