# Supplementary material for: A Comparative Assessment of Non-Laboratory-Based versus Commonly Used Laboratory-Based Cardiovascular Disease Risk Scores in the NHANES III Population
Source: PLoS One. 2011 May 31;6(5):e20416. doi: 10.1371/journal.pone.0020416 (PMC3105026; doi:10.1371/journal.pone.0020416)
Supplement: Appendix S4 — Risk discrimination results for four laboratory-based risk scores, each compared to non-laboratory-based risk score-CVD death. (DOC) [file pone.0020416.s004.doc]

Appendix S4. Risk discrimination results for four laboratory-based risk scores, each compared to non-laboratory-based risk score - CVD death

| MEN (full population, n=6,273) | | | | | | | |
| --- | --- | --- | --- | --- | --- | --- | --- |
| score | c-statistic (95% CI), un-weighted | p-value* | c-statistic, weighted** | sensitivity*** | specificity*** | PPV*** | NPV*** |
| Non-laboratory-based | 0.812 (0.779, 0.844) | -- | 0.803 | 0.785 | 0.670 | 0.062 | 0.991 |
| Framingham CVD (2008) | 0.810 (0.778, 0.841) | 0.76 | 0.801 | 0.792 | 0.670 | 0.062 | 0.991 |
| Framingham CVD (1991) | 0.814 (0.784, 0.845) | 0.65 | 0.803 | 0.795 | 0.670 | 0.063 | 0.992 |
| SCORE high risk | 0.809 (0.777, 0.841) | 0.57 | 0.804 | 0.802 | 0.669 | 0.063 | 0.992 |
| SCORE low risk | 0.809 (0.777, 0.841) | 0.57 | 0.804 | 0.796 | 0.668 | 0.063 | 0.992 |
| WOMEN (full population, n=6,958) | | | | | | | |
| Non-laboratory-based | 0.819 (0.784, 0.852) | -- | 0.819 | 0.578 | 0.860 | 0.080 | 0.990 |
| Framingham CVD (2008) | 0.847 (0.812, 0.870) | *0.004* | 0.847 | 0.621 | 0.861 | 0.086 | 0.991 |
| Framingham CVD (1991) | 0.833 (0.798, 0.862) | 0.11 | 0.833 | 0.607 | 0.861 | 0.084 | 0.990 |
| SCORE high risk | 0.805 (0.767, 0.841) | *0.02* | 0.805 | 0.592 | 0.859 | 0.081 | 0.990 |
| SCORE low risk | 0.805 (0.766, 0.841) | *0.01* | 0.805 | 0.586 | 0.859 | 0.080 | 0.990 |
| **Difference in c-statistic compared to non-laboratory-based score, using un-weighted data* | | | | |  |  |  |
| **Italics indicate p-value < 0.05* | |  |  |  |  |  |  |
| ***Standard errors not available for weighted results* | | |  |  |  |  |  |
| ****using 10-year CHD risk >10% threshold, un-weighted data* | | |  |  |  |  |  |
| Abbreviations: Positive predictive value (PPV), negative predictive value (NPV) | | | | |  |  |  |
